# Plasma in the PICU: why and when should we transfuse?

**DOI:** 10.1186/2110-5820-3-16

**Published:** 2013-06-02

**Authors:** Sonia Labarinas, Delphine Arni, Oliver Karam

**Affiliations:** 1 Pediatric Critical Care Unit, Geneva University Hospital, 6 rue Willy Donzé, Geneva 1211, Switzerland

**Keywords:** Plasma transfusion, Plasma products, Clinical effects of plasma

## Abstract

Whereas red blood cell transfusions have been used since the 19th century, plasma has only been available since 1941. It was originally mainly used as volume replacement, mostly during World War II and the Korean War. Over the years, its indication has shifted to correct coagulation factors deficiencies or to prevent bleeding. Currently, it remains a frequent treatment in the intensive care unit, both for critically ill adults and children. However, observational studies have shown that plasma transfusion fail to correct mildly abnormal coagulation tests. Furthermore, recent epidemiological studies have shown that plasma transfusions are associated with an increased morbidity and mortality in critically ill patients. Therefore, plasma, as any other treatment, has to be used when the benefits outweigh the risks. Based on observational data, most experts suggest limiting its use either to massively bleeding patients or bleeding patients who have documented abnormal coagulation tests, and refraining for transfusing plasma to nonbleeding patients whatever their coagulation tests. In this paper, we will review current evidence on plasma transfusions and discuss its indications.

## Review

### What is plasma?

Plasma units are biological products containing the acellular portion of blood obtained from a whole-blood donation after centrifugation or by plasmapheresis. Most plasma units contain 200 to 250 ml, but plasmapheresis-derived units may contain as much as 400 to 600 ml.

Plasma has been available for transfusion since 1941 and was initially mainly used as volume replacement [1]. Its use rapidly spread in the context of World War II and the Korean War for the treatment of traumatic shock [2]. With emergence of crystalloids and purified albumin, as well as with the recognition of hepatitis transmission and transfusion related side effects, its use as volume expander has progressively declined.

### Plasma constitution

Plasma units contain many biologically active molecules [3]:

– Proteins: 5.5 g/dl

○ 60%: albumin

○ 5%: coagulation factors: 1 ml of plasma contains approximately 1 unit of each of the coagulation factors, with some heterogeneity [4].

○ Fibrin

○ Immunoglobulins

○ Antithrombin, Protein C and S

– Other proteins: cytokines

– Electrolytes: Glucose (535 mg/dl), sodium (172 mEq/l), potassium (15 mEq/L) [5]

– Citrate (for stabilisation).

### Plasma products

Labile coagulations factors, especially factors V and VIII, are unstable because they have a short half-life once the unit is thawed [6]. Plasma must therefore be rapidly frozen after blood donation at −18°C or below, in order to maintain the activity of labile coagulation factors. Different products can be manufactured:

**Fresh-frozen plasma (FFP):** Plasma frozen at −18°C within 8 hours of collection, which can then be stored for up to 1 year.

**Frozen plasma (FP):** Plasma frozen within 24 hours of collection. All the clotting factors are at the same level as in FFP except for lower factor VIII (65–80% of normal) [6,7]. These levels are deemed adequate for haemostasis.

**Pooled fresh-frozen plasma:** As there is a high variability in coagulation factors between single donor units of FFP [3], pooling plasma from many donors (of identical ABO blood group) provides a method to maintain a constant concentration of these factors. However, pooling donors increases the risks of transmission of infectious diseases. To reduce this risk, multiple pathogen inactivation methods are used, such as solvent/detergent (SD) treatment, methylene blue (MB), ultraviolet light with riboflavin, and psoralens (amotosalen). SD-FFP inactivates enveloped virus but has no effect on hepatitis A virus and parvovirus B19. SD-FFP also seems to reduce HLA concentrations and bioactive lipids, which could lead to fewer immunologic adverse effects [8].

MB-FFP has low factor VIII (FVIIII) and fibrinogen activity, whereas SD-FFP has reduced activity of VWF et FVIII as well as lower functional activity of protein S. Therefore, SD-FFP should not be used in patients with IgA or severe protein S deficiency. However, although FFP and SD-FFP differ in several aspects [9], they are considered to be interchangeable in most European countries.

**Thawed plasma:** FFP or FP thawed at 37°C (if thawed at 4°C, cryoprecipitate will form) and then stored for up to 5 days at 1 to 6°C [10]. The advantage of thawed plasma is its availability for immediate use. It therefore can be used rapidly, for example in massively bleeding patients. Nevertheless, it should not be used if either factor V or factor VIII is specifically needed because of the decline of their activity [1]. Furthermore, its usefulness and safety has not been demonstrated yet in neonates and infants.

**Cryoprecipitate:** Cryoprecipitate is prepared by thawing frozen plasma at 1°C to 6°C, which causes some proteins to precipitate. This cryoprecipitate is rich in fibrinogen, factor VIII, factor XIII, and von Willebrand factor (vWF). It is then refrozen at −18°C and can be stored up to 1 year.

## What are the clinical effects of plasma?

### What is the effect of plasma transfusions on coagulation test results?

As plasma contains coagulation factors [3], its most frequent indication is to correct abnormal coagulation tests [1]. In massively bleeding patients, large-volume, crystalloid administration followed by RBCs alone promotes dilutional coagulopathy. Furthermore, active bleeding leads to coagulation factor consumption. Development of shock and coagulopathy early after injury is strongly associated with mortality [11]. Plasma transfusions can constitute a life-saving intervention by improving coagulation factor deficit [12]. Indeed, plasma transfusions have been shown to partially correct very abnormal international normalized ratio (INR) levels [13].

In less dramatic situations, the impact of plasma transfusions on coagulation tests has only been explored since 2006, when Abdel-Wahab et al. assessed the effect of plasma on INR in an adult critical care unit [14]. Among 121 critically ill adults who received plasma for moderately abnormal coagulation tests (INR < 1.85), only one (0.8%) was able to correct his INR (<1.1) after a plasma transfusion. The amount of transfused plasma was not associated with the INR after transfusion, whatever the volume. The authors concluded that, whatever the volume, plasma transfusions did not correct moderate coagulopathy. The same year, this was confirmed in another observational study by Holland et al., who showed that plasma transfusions did not correct INR levels < 2.0–2.5 [13].

It should be noted that no study has evaluated the effect of plasma transfusions on coagulation test results in children. Therefore, observational data suggest that plasma transfusions improve outcome in massively bleeding patients but fails to do so in nonmassively bleeding patients with mildly abnormal coagulation tests.

### How does plasma affect the immune system?

As plasma contains many bioactive substances, such as cytokines, it has been presumed that it could induce notable changes in the patient’s immunity status. This transfusion-associated immunomodulation (TRIM) has been described in all blood product transfusions. TRIM effects may be mediated by: 1) allogeneic mononuclear cells; 2) white-blood-cell (WBC)-derived soluble mediators; and/or 3) soluble HLA peptides circulating in allogeneic plasma [15]. Prestorage leucocyte depletion has been shown to prevent TRIM [16]. Nevertheless, in vitro studies have revealed that even leuco-depleted plasma increased the levels of tumor necrosis factor (TNF) and interleukine-10 (IL-10) after transfusion [17]. One might hypothesize that this is due to the freezing process, which ruptures the cell walls in the remaining WBC, as leukoreduction leaves < 1×10^6^ WBC/unit. Other studies also have described the different effects of filtration of interleukins, histamine, and TNF in filtrated blood components [18]. However, clinically relevant concentrations of these substances have never been determined [19].

All of these *in vitro* studies suggest that plasma is biologically active and that it might induce immune modifications in the recipient. However, the specific clinical impact of these substances has still to be assessed *in vivo*.

## What are the clinical adverse effects of plasma?

### Infections

As for all other transfusions, plasma also is associated with transfusion-transmitted infectious diseases. However, plasma transfusions are associated with a much lower risk of bacterial infections (<0.1/100,000) than RBCs (0.4/100,000) and platelets (2.9/100,000) [20]. This might be due to the freezing process *per se*, which inactivates bacteria.

Apart from donor screening (Table 1), multiple pathogen inactivation methods have been developed to reduce this risk, especially for pooled plasma. Furthermore, removal of cellular components also eliminates cell-associated bacteria, as well as most protozoa and cell-associated virus. However, freezing does not remove free viruses, such as hepatitis A, B, and C, human immunodeficiency virus (HIV) 1+2, parvovirus B19, and prions. Table 2 shows the incidence of some viral transmission risks for FFP and SD-FFP compared with RBC units.

**Table 1:** Common safety requirements for FFP donors

**Blood test**	
HIV 1+2	Negative
HbsAg (surface antigen of hepatitis B virus)	Negative
HCV antibody (hepatitis C virus antibody)	Negative
HCV genome	Negative
Treponema pallidum antibody	Negative
Alanine aminotransferase (ALT)	Negative

**Table 2:** **Incidence of infectious disease transmission risks, based on Heiden et al. **[18]** and on Stramer **[21]

	**FFP**	**SD-FFP**	**RBC**
HIV 1+2	1:10 million	No reported cases to date.	1:2.1 million
Hepatitis C	1:50 million	No reported cases	1:1.9 million
Hepatitis B	1:1–2 million	No reported cases	1:205 000–488 000

Transfusion-related acute lung injury:

Transfusion-related acute lung injury (TRALI) is defined as a noncardiogenic pulmonary oedema related to any transfusion therapy. Clinical features may be indistinguishable from other causes of acute lung injury (respiratory distress, hypoxia, and pulmonary infiltration). As a result, this complication is perhaps underdiagnosed and underreported. Most TRALI symptoms occur within 1 to 2 hours after transfusion initiation, and almost all reactions occur by 6 hours after transfusion completion. The mechanisms implicated consist of passive transfer of antileukocyte antibodies (anti-human leucocyte antigen [HLA]) from alloimmunized donors and the accumulation of bioactive mediators in a predisposing inflammatory condition.

Many adult studies have shown that among blood products, plasma and platelet transfusions were the most frequently involved in TRALI [22-24]. The UK hemovigilance analysed 3,239 reports of adverse reactions and events associated with transfusions of labile blood components in the UK, between 1996 and 2005. Twenty of the 185 cases of TRALI happened in paediatric patients. In 19 of these cases (95%), the implicated component was either plasma or platelets [25].

### Immunomodulation

As already discussed, plasma contains many bioactive substances, such as cytokines, immunoglobulin, and coagulation factors. Transfusion-related immune modulation might explain why standard plasma transfusions seem to be associated with some adverse events, such as multiple organ dysfunction syndromes and an increased risk of contracting nosocomial infections. Adult epidemiological studies have shown that patients who are transfused with plasma are indeed more prone to nosocomial infections (adjusted odds ratio 2.99 (95% CI 2.28–3.93)) [26] and acute respiratory distress syndrome (adjusted odds ratio 2.48 (95% CI 1.29; 4.74)) [22].

A paediatric retrospective study evaluated the association between plasma and mortality in 380 children with acute lung injury (ALI) [27]. The authors showed a significant increase in mortality (8%) for each ml/kg of FFP transfused in paediatric patients with acute lung injury, after adjusting for severity at admission and coagulopathy. We recently published a prospective epidemiological study in 831 critically ill children. The adjusted odds ratios were 3.2 (95% CI 1.6; 6.6) and 2.3 (95% CI 1.0; 5.3) for increased incidence morbidity and nosocomial infection, respectively. There also was a significant difference in the adjusted length of stay in the intensive care unit [28]. Although these nonrandomized studies might be biased, plasma transfusions may be associated with worse clinical outcome, perhaps due to their immunomodulative properties.

### Allergic reactions

Real anaphylaxis with the administration of plasma is very rare. In the UK, there were only 23 allergic and 25 anaphylactic reactions imputable to plasma transfusions, during a 6-year period [1]. In France, in 2010, allergic reactions occurred in 0.05% of all plasma transfusions [20].

### Other reactions

Plasma transfusions are associated with transfusion-associated circulatory overload (TACO). TACO occurs when the recipient’s circulatory system is overwhelmed by either the rate of the infusion or the volume of blood products transfused. TACO is characterized by the acute onset of dyspnea and typically hypertension, tachypnea, and tachycardia. A significant increase of brain-type natriuretic peptide (BNP) above the recipient’s baseline level can help to differentiate TACO from TRALI. The exact prevalence of TACO is unknown: the proportion of adults requiring plasma transfusions who develop TACO ranges from 0.001% to 4.8% of [20,29]; there is no specific paediatric data.

Plasma transfusions also have been associated with citrate toxicity, which have been described after rapid transfusion of large volume of plasma [30]. Blood components are anticoagulated with citrate. When transfused, citrate may bind with circulating ionic calcium and magnesium. During massive transfusion, the capacity of the liver to metabolize citrate may be overwhelmed, and potentiated by hypothermia or hypotension, particularly in the presence of underlying liver disease, leading to hypocalcaemia and/or hypomagnesemia. Metabolic alkalosis also may develop secondary to the accumulation of bicarbonate, the metabolic byproduct of citrate.

### What is the current practice for plasma transfusions?

In 2008, according to the U.S. Department of Health and Human Services, 4,484,000 plasma units were transfused in the United States [31]. Close to 3% of all paediatric admissions [32] and more than 10% of critically ill children [28] receive a plasma transfusion during their stay, making plasma transfusions a frequently used treatment modality.

When physicians prescribe plasma transfusions, they usually hope to correct abnormal coagulation tests by restoring coagulation factor levels, either with a therapeutic (to stop bleeding) or prophylactic (before invasive procedures or surgery) goal. It seems that the majority of plasma is administered in the latter circumstance. In 2004, Dzik et al. described that the most common purpose of plasma transfusion was to “prepare” a patient with an elevated international normalized ratio (INR) for invasive procedures [33]. Lauzier et al. also showed that plasma transfusions often were administered to patients who were not bleeding [34]. In another prospective study, Arnold et al. showed that 60% of the plasma was prescribed to either nonbleeding patients or patients with normal coagulation tests [35]. In another audit, Shariff et al. found that 34% of the use of plasma units in their centre was inappropriate and concluded that this was “due to limited knowledge of its use in specific situations and ignorance of risks” [36].

We recently presented the only paediatric data regarding current practice. More than two thirds of paediatric intensivists would prescribe plasma transfusions to nonbleeding children. For these nonbleeding critically ill children, the median INR level that would trigger a plasma transfusion was 2.0 to 2.5, according to the clinical situation [37].

This heterogeneity in plasma transfusion practice patterns probably indicates a need for stronger evidence on appropriate plasma transfusion thresholds. Once randomized, controlled trials will have provided the clinicians with the best transfusion strategies, these will have to be implemented. It has been shown that systemic organizational interventions, such as educational campaigns or prospective audits of plasma requests, lead to a significant reduction in the inappropriate rate of plasma transfusions [38].

### How and when to transfuse plasma in the PICU?

The following recommendations are based on international guidelines [1,30,39,40].

### Appropriate trigger

Because it has been shown that plasma transfusions fails to correct INR < 1.85 or 2.0 [13,14], most recommendations advise to transfuse if PT ratio or aPTT ratio >1.5 [1,30] or if INR >2 [40]. Thromboelastography, a recently incorporated method into ICU clinical practice, is performed with whole blood. It assesses the viscoelastic property of clot formation under low shear condition [41]. Thromboelastography-based algorithms reduce both transfusion requirements and blood loss in massively bleeding patients [42]. There is conflicting data regarding the predictive value of thromboelastography tests in patients with liver disease [43,44]. However, there is currently no data on its adequacy to evaluate the risk of bleeding in other situations, especially in a PICU setting. Therefore, it has not yet been incorporated in plasma transfusion guidelines.

Physicians also must recognise that coagulation tests are not well correlated to the risk of bleeding. In a recent meta-analysis of 25 studies, Segal et al. have shown that the risk of bleeding during different procedures (angiography, liver biopsy, thoracocentesis, bronchoscopy, liver laparoscopy, etc.) was similar for patients with normal and abnormal coagulation [45].

### Appropriate dose

Hemostasis can be achieved when activity of coagulation factors is at least 25-30% of normal, because there is a nonlinear, exponential relationship between clotting factor levels and coagulation test results [46]. In adults, because the total blood volume is approximately 65 ml/kg and the hematocrit is approximately 0.4, the plasma volume (i.e., the volume without RBC) is approximately (1–0.4) × 65 ml/kg = 40 ml/kg. Therefore, to provide 30% of coagulation factor, the therapeutic transfusion dose theoretically should be 30% × 40 ml/kg = 12 ml/kg. Most experts suggest transfusing 10 to 15 ml/kg; however, no clinical trial has evaluated this dose.

### Prescription of plasma transfusion

Compatibility testing is not required before plasma transfusion, unless large volumes are given. Plasma must be ABO compatible with the recipient’s RBCs. Type AB plasma can be administered in severe and acute situations as a universal donor type if necessary. Rh compatibility and cross-matching are not considered because there are virtually no RBCs in FFP.

Normally, it takes 20 to 30 minutes to thaw FFP. Plasma also can be warmed in 7 minutes using a microwave oven; however, only ovens specifically constructed for this task can be used, as standard microwaves will destroy the function of most coagulation factors.

Thawed plasma must probably be used within 4 to 6 hours after release. A macroaggregate filter must be used to avoid infusing microcrystals of unthawed plasma.

### Prophylactic indications

Current recommendations advise against the use of plasma for volume expansion, as it has been show that colloids are not superior to crystalloids [47], and plasma transfusions are associated with a worse clinical outcome [28]. Because a recent systematic review of 80 randomized, controlled trials did not find any significant benefit of prophylactic use of plasma transfusions [48], a prophylactic plasma transfusion strategy is not supported. However, most experts advocate prophylactic plasma transfusion in case of surgery or invasive procedures in patients with abnormal coagulation tests.

### Therapeutic indications

#### Massive transfusions

Patients who are massively bleeding (transfused between 40–80 ml/kg of RBCs in a 24-hour period) have dilutional coagulopathy as well as coagulation factor consumption that might be corrected with plasma transfusions. Most experts suggest not waiting for coagulation tests before transfusing plasma units. The optimal RBC:plasma:platelet ratio is still unknown, but early use of plasma and platelets seems to be associated with improved outcome [49]. The use of rapid thromboelastography testing to guide plasma transfusion in massively bleeding patients, while still uncommon, is increasing over time. Thromboelastography-based algorithms reduce both transfusion requirements and blood loss in cardiac surgery, liver transplantation, and massive trauma.

#### Bleeding patients

In nonmassive bleeding patients, expert consensus is that plasma should be considered only if the PT ratio or aPTT ratio are > 1.5 [1,30] or if INR is >2 [40]. No recommendations in this population currently include thromboelastography thresholds for plasma transfusion.

#### Acute disseminated intravascular coagulation (DIC) in conjunction with active bleeding

In bleeding patients with DIC, some physicians give 10–15 ml/kg of plasma, in association with the correction of the underlying cause. However, this strategy has never been adequately evaluated.

#### Liver disease

In children undergoing liver transplantation, plasma transfusions are associated with increased 1-year mortality [50]. Therefore, it seems reasonable to transfuse plasma only in the setting of a severe bleeding with abnormal coagulation tests.

#### Surgical or invasive procedure

Plasma also is often used to try to prevent bleeding before surgical procedures or invasive procedures in patients with abnormal coagulation tests. It has been shown that abnormal coagulations tests are not correlated with an increased risk of bleeding [45]. However, there are no data regarding this transfusion strategy.

#### Single clotting factor deficiencies

In patients with single clotting factor deficiency and active bleeding, experts recommend that plasma should only be transfused if specific concentrate is not obtainable. Usually, this only applies to factor V deficiency.

#### Reversal of warfarin effect

Warfarin causes a functional deficit of factors II, VII, IX, and X, as well as proteins C and S. Plasma should only be used for the reversal of warfarin if there is evidence of severe bleeding or intracranial haemorrhage. If available, prothrombin complex is the first-choice treatment.

#### Hereditary angioedema (Quince oedema)

The purpose of treatment of acute episodes is to halt progression of the oedema as quickly as possible. The most efficient treatment is C1 inhibitor concentrate from donor blood, which must be administered intravenously. If C1-esterase inhibitor is not available, plasma transfusions can be used to treat these patients.

#### Haemorrhagic disease of the newborn

In neonates, coagulation test have a wider normal range than in adult and therefore are even less correlated to the risk of bleeding. As for older children, there are no data to recommend prophylactic plasma transfusions. In case of significant bleeding, some experts recommend giving plasma (15 ml/kg) and intravenous vitamin K.

### Current recommendations *against* the use of plasma

Plasma should not be used as volume replacement, because there is no advantage of colloids over crystalloids for this indication [47].

Plasma should not be used to correct abnormal INR or PTT in nonbleeding patients who have no planned surgery or invasive procedures, because there is no correlation between coagulation tests and risk of bleeding [45], but data show that plasma transfusions increase the risk of unfavourable outcome [27,28].

### Future directions

There are many unresolved questions regarding plasma transfusions. Apart from massively bleeding patients, it is still unknown which patients really benefit from plasma transfusions. Is it appropriate to give prophylactic plasma transfusions to patients with abnormal coagulation tests who will undergo surgery? Should we transfuse plasma to patients who experience minor bleeding? Randomized, controlled trials on plasma transfusion strategies in these different clinical situations are warranted.

Currently, it is unclear which coagulation test best reflects the risk of bleeding. Therefore, it is still unclear what the appropriate plasma transfusion triggers should be. Some experts suggest that thromboelastography tests are superior to classical coagulation tests. However, no studies have yet shown that a thromboelastography-based goal-directed transfusion strategy improves mortality in critically ill children.

## Conclusions

Plasma transfusion is a common treatment for critical care patients. Although it clearly benefits some patients, such as those who are massively bleeding, epidemiological studies suggest that, in less dramatic situations, plasma transfusions are associated with worse outcome, both in adults and children. Therefore, the decision to proceed with plasma transfusion must be based on individualized indications, while balancing the risks and benefits.

Unfortunately, no randomized, controlled trial has yet addressed the appropriate plasma transfusion threshold. Despite current expert recommendations based on observational data, most physicians prescribe plasma transfusions according to their own believe and experiences. This probably leads to many unnecessary transfusions and therefore preventable adverse events.

## Abbreviations

ALI: Acute lung injury; aPTT: Activated partial thromboplastin time; ARDS: Acute respiratory distress syndrome; DIC: Acute disseminated intravascular coagulation; FFP: Fresh-frozen plasma; FP: Frozen plasma; HIV: Human immunodeficiency virus; HLA: Human leucocyte antigen; IL-10: Interleukine-10; INR: International normalized ratio; PRP: Pathogen reduced plasmas; PT: Prothrombin time; TNF: Tumor necrosis factor; TRALI: Transfusion-related acute lung injury; TRIM: Transfusion-associated immunomodulation; TTP: Thrombotic thrombocytopenic purpura; vWF: von Willebrand factor.

## Competing interests

All of the authors declare they have no competing interests.

## Authors’ contributions

SL conceived the review. DA and OK reviewed and modified the draft, and completed the final version. All authors read and approved the final manuscript.
